# Big Data Collection in Large-Scale Wireless Sensor Networks

**DOI:** 10.3390/s18124474

**Published:** 2018-12-18

**Authors:** Asside Christian Djedouboum, Ado Adamou Abba Ari, Abdelhak Mourad Gueroui, Alidou Mohamadou, Zibouda Aliouat

**Affiliations:** 1 LaRI Lab, University of Maroua, P.O. Box 814 Maroua, Cameroon; asside.djedouboum@univ-maroua.cm (A.C.D.); alidou.mohamadou@univ-maroua.cm (A.M.); 2 Faculty of Exact and Applied Sciences, University of Moundou, P.O. Box 206 Moundou, Chad; 3 LI-PaRAD Lab, Université Paris Saclay, University of Versailles Saint-Quentin-en-Yvelines, 45 Avenue des Etats-Unis, 78000 Versailles, France; mourad.gueroui@uvsq.fr; 4 LRSD Laboratory, University Ferhat Abbes Setif 1, El Bez, Setif 19000, Algeria; zaliouat@univ-setif.dz

**Keywords:** Wireless Sensor Networks, data collection, Big Data, IoT

## Abstract

Data collection is one of the main operations performed in Wireless Sensor Networks (WSNs). Even if several interesting approaches on data collection have been proposed during the last decade, it remains a research focus in full swing with a number of important challenges. Indeed, the continuous reduction in sensor size and cost, the variety of sensors available on the market, and the tremendous advances in wireless communication technology have potentially broadened the impact of WSNs. The range of application of WSNs now extends from health to the military field through home automation, environmental monitoring and tracking, as well as other areas of human activity. Moreover, the expansion of the Internet of Things (IoT) has resulted in an important amount of heterogeneous data that are produced at an exponential rate. Furthermore, these data are of interest to both industry and in research. This fact makes their collection and analysis imperative for many purposes. In view of the characteristics of these data, we believe that very large-scale and heterogeneous WSNs can be very useful for collecting and processing these Big Data. However, the scaling up of WSNs presents several challenges that are of interest in both network architecture to be proposed, and the design of data-routing protocols. This paper reviews the background and state of the art of Big Data collection in Large-Scale WSNs (LS-WSNs), compares and discusses on challenges of Big Data collection in LS-WSNs, and proposes possible directions for the future.

## 1. Introduction

### 1.1. Background

According to the United Nations, the world’s population is estimated at 7.6 billion in 2018. In addition, more than 2 billion people are regularly connected to the Internet and roughly 5 billion of the world’s population use various kinds of connected objects. During the last decade, the amount of global generated data has exploded with the increasing use of connected objects, and especially the Internet of Things (IoT), which continuously produces most heterogeneous data. Cisco reported that, by the end of 2019, the IoT will annually generate more than 500 zettabytes of structured and unstructured data, and that number is expected to exponentially grow [1]. Moreover, a number of industries’ forecasts are projecting 50 billion connected devices to the Internet by 2020 [2].

Furthermore, in recent years, with the expansion of the IoT and recent technological advancements, a growing number of intelligent information-aware devices, such as sensors, actuators, smartphones, smart wristbands, tablets, devices based on readers’ Radio Frequency Identification (RFID) and Machine-to-Machine (M2M), have led to the exponential growth of generated-data volume [3,4,5,6,7,8,9]. In general, these devices, and especially sensors, are used both in industry and research to collect data in various application contexts, such as healthcare, environmental monitoring, and precision agriculture [10]. The significant advance in research and engineering has introduced novel architectures for data acquisition, transmission, large-scale data storage, and processing. Moreover, the emerging Big Data paradigm, mainly used to describe massive data, is experiencing renewed interest in both the industrial and scientific world [11]. A number of domains, such as the IoT, artificial intelligence, and data science, are benefiting from digital transformation allowed by Big Data.

Moreover, Internet-based companies, such as Google, Twitter, Facebook, and Amazon, motivate the current thrust of Big Data research, where real-time data originating from human sources, which include e-mails, online shopping history, as well as tweets, are used in large-scale data mining and for data analysis to produce a number of value-added services [12]. Fundamentally, Big Data may be seen as a very large number of structured, semistructured, and unstructured data of a wide variety of data types that is generated with high velocity and has the potential to help us in understanding hidden information in datasets, and also in managing and organizing these datasets [13]. Usually, the Big Data process relies on four steps: data generation, acquisition, storage, and analysis. Contrary to traditional data, Big Data need more real-time analysis given the fact that they include massive amounts of unstructured data of various types.

Nowadays, IoT devices are commonly used in a large spectrum of critical application domains, including eHealth, smart environments, smart cities, smart building, as well as precision agriculture. The massive data collection enabled by IoT technology, leads to the emergence of a huge number of sensor-based applications. Given the virtual abstraction of smart objects enabled by IoT technology, Wireless Sensor Networks (WSNs) have become the most commonly used IoT-based platforms for data collection [14]. Therefore, WSNs are known to be a fundamental component that allowed the development of the IoT system. In such networks, a large number of sensors are deployed in the order of thousands, collaborating to collect and transfer information to the base station [15,16].

Increasingly, there is a growing trend towards large-scale sensor networks for data collection. Although they represent a new generation of sensor networks, their use is subjected to many constraints, including adaptability with existing methods for network scaling. Several important challenges need to be addressed in order to allow a network to effectively play its role. Among these challenges, we can mention the optimal deployment of sinks, the minimization of sensors’ energy consumption, as well as their lifetime [17,18]. In a Big Data context, with a massive amount of high-speed, regularly changing, and variable data, large-scale WSNs (LS-WSNs) may provide valuable solutions for massive data collection [19].

However, power-aware Big Data gathering in large-scale sensor networks remains, therefore, a challenging research issue. In a computer vision domain, for example, managing a large number of learning instances of images is not an easy task [20]. Nevertheless, in LS-WSNs, real-time data generation and acquisition are achieved by various sensors, such as gathering information on proximity, location, humidity, and movement. Unfortunately, data generated and acquired from individual sensors may not seem significant. However, the global various types of data generated by a number of sensors in LS-WSNs can produce significant Big Data [21]. Moreover, data collection in LS-WSNs requires two important challenges to be addressed, that is, monitoring in large geographic environments with unconnected regions, and power-aware data collection.

A number of studies that provide a background to WSNs and Big Data on the one hand, and overviews of data-collection schemes in the context of LS-WSNs on the other, have been proposed in the recent literature. We reviewed most of the latest existing papers related with the topics examined in this paper. In this light, Rawat et al. (2014) [22] proposed a review of recent developments in WSNs by surveying the leading research projects, platforms, standards, and technologies related with sensor networks. Bouaziz et al. (2016) [23] as well as Sanchez et al. (2018) [24] have reviewed recent trends of mobility management in WSNs while highlighting applications and platforms. Di Francesco et al. (2011) [25] provided an extensive survey and taxonomy of the data-collection process with mobile elements in WSNs. In addition to sensors, there has been a recent tendency to use unmanned aerial vehicles like drones for data acquisition. In this context, swarm intelligence schemes based on bacterial foraging optimization have been successfully used to collect massive data in LS-WSNs [26,27].

In 2014, Takaishi et al. [21] proposed a Big Data gathering scheme in densely deployed WSNs that exploit sink mobility. In the same year, Wu et al. [28] proposed a Big Data collection scheme based on the structure-fidelity approach. The authors provided a framework for collecting structured data and to maintain data fidelity in terms of structural similarity in continuous sensing applications in WSNs. After defining what really represents a big sensor data system, Ang et al. (2017) [29] surveyed the progress made in the development and applications of big sensor data research, especially in the context of smart cities. In order to enhance Big Data collection in an IoT system, Ang et al. (2018) [12] proposed a way to collect Big Data in LS-WSNs using mobile collectors to optimize energy consumption. The authors adopted a data-collection scheme using a date mule and mobile access point, which are the mostly used components in data-collection models in sensor networks.

According to the literature, we reviewed select research in the context of this study. We established that much less research has been done on Big Data collection with sensor-based systems compared with standard Big Data systems. Standard Big Data technology using stored data is subject to challenges related to the speed capacities of communication networks. Useful technologies, such as Hadoop or MapReduce, combined with cloud-computing technology are actually used to address communication networks by integrating distributed processing.

Certainly, future 5G systems, which include a promising cloud-based radio access network model and a more powerful mmWave spectrum, will be more flexible, agile, converged, and useful for automating data analytics with machine-learning or swarm-intelligence algorithms. However, unstructured large volumes of data gathered from IoT-based systems, such as LS-WSNs, need more attention. The focus of this paper is twofold. Firstly, the potential of WSNs and Big Data is studied, with particular emphasis on data collection. Then, existing data-collection schemes in the literature are reviewed and discussed. This paper gives a global overview of Big Data collection in LS-WSNs with the challenges to be addressed. Therefore, the aim of this paper is to provide useful insights to research and industry into this promising research area, and motivate the development of new schemes, models, and frameworks for Big Data collection in LS-WSNs.

### 1.2. Author’s Contributions

This work aims at increasing readers’ knowledge by providing a sufficient and comprehensive background in the schemes, algorithms, and models used for resolving different data-collection challenges in LS-WSNs. The first contribution of this paper is enabling novice readers to understand the mechanism and operation of large-scale wireless sensor networks for massive data collection. Secondly, we propose an architecture for a WSN dedicated to Big Data collection. Then, we lay the groundwork for our future work, which consists on the one hand of proposing a deployment model of large-scale WSNs, which should maximize coverage, and, on the other hand, proposing efficient and lightweight algorithms for Big Data collection in LS-WSNs.

### 1.3. Organization of the Paper

The rest of this paper is organized as follows. We first provide an overview of WSNs in Section 2. We then propose a background on Big Data in Section 3. Section 4 focuses on Big Data collection in LS-WSNs. In Section 5, we reviewed data transferring schemes in the context of LS-WSNs. This is followed in Section 6 by a discussion on the challenges of Big Data collection in LS-WSNs. Finally, we conclude the paper in Section 7.

## 2. Overview of WSNs

**Figure 1:**
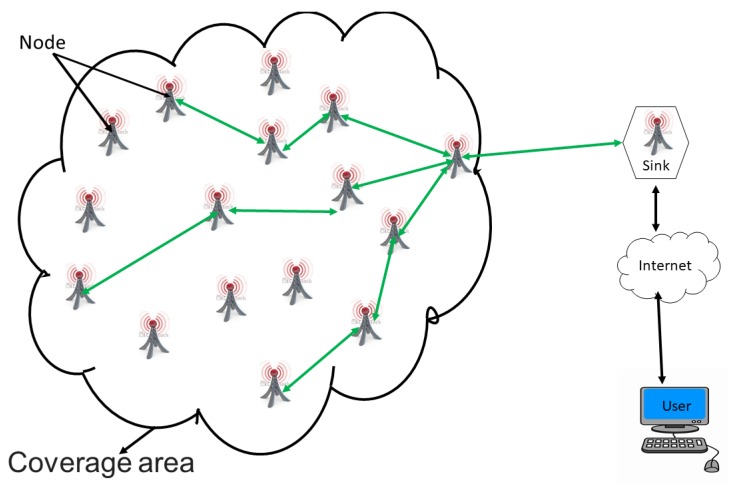
General architecture of a wireless sensor network (WSN).

A WSN is an ad hoc network consisting of a set of sensor nodes, randomly fixed or dispersed in a given geographical area, communicating via a wireless link to autonomously collect, process, and transmit data in their environment to a special node, considered the collection point, called a sink [16,22,30,31,32]. The geographical area where the sensor nodes operate is called the field or area of interest. In a WSN, sensor nodes are able to self-organize to collect information about the environment where they are deployed. The transmission of the collected data can be done periodically or event-based, depending on the nature of the implemented application. The sink is a node with two or more network interfaces that act as a bridge between the WSN and the end-user’s network (for example, a local area network or the Internet). The user can send requests to other nodes in the network via the sink, for example, to specify the type of data to be collected. For illustration purposes, Figure 1 shows the basic architecture of a WSN. The sensor nodes randomly deployed in a zone of interest and the sink located at the end of the zone are responsible for recovering the data collected by the sensors. Most often, the sink collects and processes network data, thus sending only relevant information to the user. It can also receive commands to run on the internal network from it (the user). The collected data are processed and analyzed by the user.

### 2.1. Hardware Architecture of a Wireless Sensor

**Figure 2:**
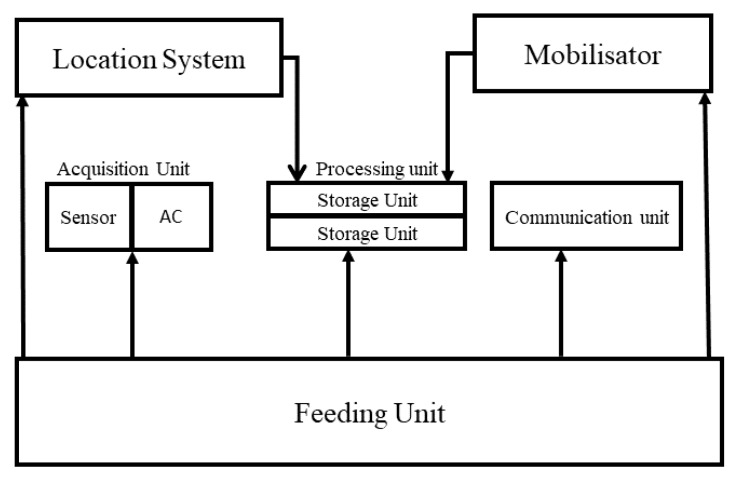
Architecture of a wireless sensor.

A wireless sensor is a small electronic device capable of measuring a physical quantity (e.g., temperature, light, pressure) and communicating it to a collection center directly or via other sensor nodes that act as routers [10]. Given the advances in microelectronics, wireless transmission technologies and software have made it possible to produce microsensors of a few cubic millimeters in volume that can operate in a network at a reasonable cost [22]. A sensor node consists of four base units and two optional units [10,33] (see Figure 2):

- *Acquisition unit*. This consists of two subunits: a sensor and an analog-to-digital converter (ADC). The sensor collects digital measurements on environmental parameters and transforms them into analog signals. The ADC converts analog signals into digital signals.
- *Processing unit*. This is also composed of two units: a data-storage unit, and a processor responsible for data-processing and control procedures allowing the collaboration of the sensor with the others in order to carry out acquisition tasks.
- *Communication unit*. Its function is information transmission and reception. It is equipped with a transmitter/receiver pair. It allows communication within the network, in our case, by radio frequency (radio waves). However, there are other transmission possibilities (e.g., optical and infra red)
- *Power unit*. Power supply is a critical element of sensor architecture. It is responsible for supplying energy to all other units. It most often corresponds to a battery, feeding the sensor, whose limited resources make it a problem specific to this type of network. However the recent realization of solar-panel power unit is trying to provide a solution for extending sensor life [33].
- *Mobilizer*. Optionally, this is used to move the node to complete the task to be processed.
- *Location-Finding System*. Optionally, this provides information on the location required by the application and/or routing.

### 2.2. Types of WSNs

**Figure 3:**
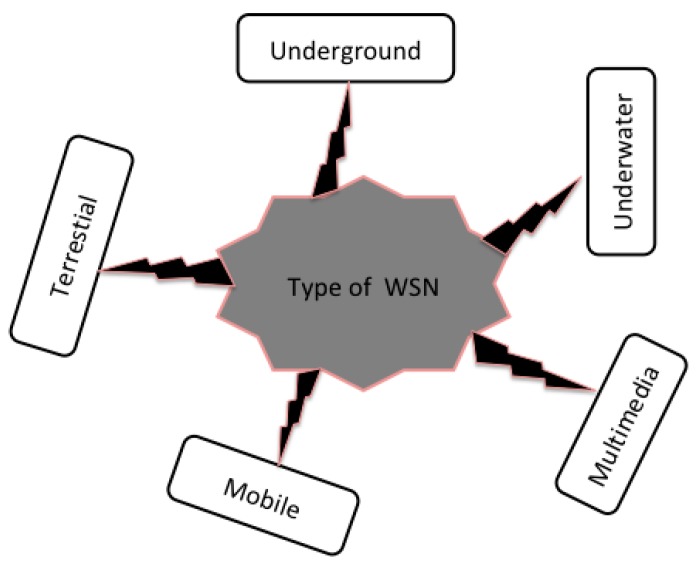
Types of WSNs.

Depending on the deployment environment on land, underground, or underwater, there are several types of WSNs [10] (see Figure 3):

- *Terrestrial*. This kind of WSNs is intended to be deployed in a terrestrial space. In these networks, hundreds of thousands of sensors are deployed randomly or deterministically over a given area. This type of WSN is much more useful in the field of environmental monitoring and natural phenomena; however, it represents a challenge for the sustainability of the network in terms of energy management [34].
- *Underground*. These networks consist of very special sensor nodes known for their high cost and the logistics necessary for their implementation and maintenance. In this type of network, the sensor nodes are installed in the ground. They find their application in agriculture or in minefields, where they are used to monitor conditions in the soil. However, in this type of network, there is a ground node that has a role in transmitting the information detected by the underground nodes to the base station [35].
- *Underwater.* This particular type of WSNs is networks hidden under the sea. Because of the hostility represented by the environment of their deployment, this type of network remains an interesting research challenge. These nodes are more expensive than terrestrial sensors, wireless communication is acoustic, bandwidth is limited, signal loss is recurrent, and propagation delays and synchronization problems are frequent [36,37].
- *Multimedia*. These WSNs are designed to monitor and trace data such as images, videos, and sound. These sensors are equipped with cameras and microphones. This type of network requires good bandwidth and good quality of service, which implies high power consumption for data processing and compression. Preplanning is required for the deployment of these sensors [38].
- *Mobile.* This is the most recent type of WSN, and it uses mobile nodes that can autonomously reposition and reorganize the network. After initial deployment, the nodes disperse to gather information. There is also a hybrid network consisting of a combination of mobile and fixed sensors [12,25,26,37,39,40,41,42].

### 2.3. Topologies of WSNs

Usually, an LS-WSN is composed of a large number of high-density nodes deployed in a detection area, with one or more base stations within or near the area. The base station communicates with the sensors via requests or commands, and the sensors communicate with each other via wireless communications. They participate in performing data-detecting and -sending tasks to the base station. In sensor networks, the energy consumed for communication is much higher than that required for detection or computing. For example, the transmission of a single bit of data is equivalent to 800 instructions [43]. A wireless sensor network can be organized according to different topologies, including star, mesh, and hybrid topologies.

#### 2.3.1. Star Topology

In this communication topology, the network consists of a single station that can send and/or receive messages from a number of remote sensor nodes. Sensor nodes are not allowed to communicate with each other. This topology has the advantages of being easy to deploy, and guaranteeing communication and low latency between remote sensor nodes and the base station [44].

#### 2.3.2. Mesh Topology

This topology uses a multihop network scheme to cover all connected sensor nodes, that is, the signal goes from one sensor node to another until it reaches the base station. The communication range of each node is not limited by distance; we can, however, add an intermediate sensor node to extend it [45]. The main advantage of this topology is that it maintains connectivity in the network, but its major disadvantage lies in the number of jumps that increase from one communication to another, increasing energy consumption in the network.

#### 2.3.3. Hybrid Topology

Hybrid topology combines both the star and mesh topologies [45]. It provides a robust network with several communication functions while keeping sensor energy consumption to a minimum. Sensor nodes that have higher energy capacities are enabled to provide multihop communications, while low power nodes are disabled.

### 2.4. WSN Communication

A sensor node can communicate directly, without detour, thanks to single-hop communication with the sink, or using multihop communication, passing the information to its neighbors [33].

#### 2.4.1. One-Hop Communication

**Figure 4:**
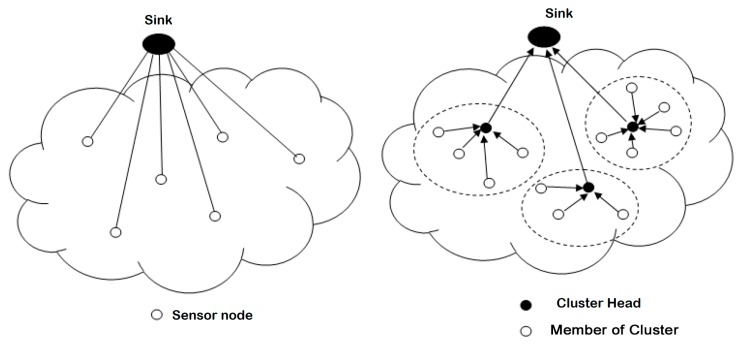
One-hop communication.

One-hop communications lead to transmissions over long distances resulting in a significant increase in energy consumption. Figure 4 illustrates this type of communication.

#### 2.4.2. Multihop Communication

**Figure 5:**
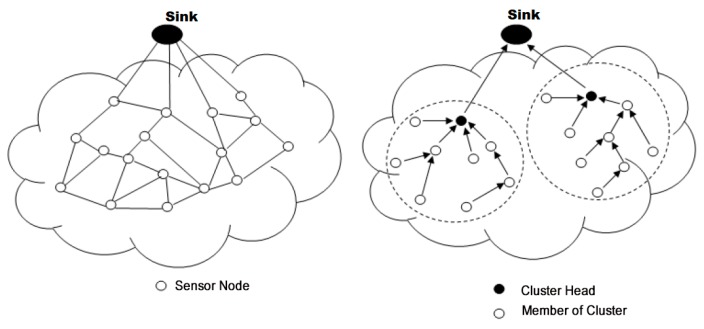
Multihop communication.

Using multihop communication reduces transmission distance, thus reducing energy loss in order to increase the service life of the network. In multihop transmissions, network architecture plays a major role. Multihop network architectures are typically divided into flat or hierarchical architectures. Figure 5 illustrates this type of communication.

### 2.5. Specific Characteristics of Sensor Networks

Sensor networks have intrinsic characteristics, both at the sensor nodes (energy power, processing capacity, storage capacity, and reach), and at the level of the network constituted by these sensors (bandwidth, density, and network topology) [33].

#### 2.5.1. Sensor-Node Features

Individually, sensor nodes have their own characteristics that usual sensors do not have. Indeed, sensor nodes have energy autonomy, and this resource is essential to the survival of a WSN because, in most cases, the sensor’s battery is irreplaceable. In addition, sensors have limited capacity, such as with regard to storage and data processing, as well as narrow sensor transmission range. Here, we detail the main features. More information is available in the literature [22].

- *Energy consumption.* Sensors have energy autonomy, and they usually use tiny batteries as energy resources. In most cases, WSNs are deployed in hard-to-reach areas. This makes it difficult or almost impossible to recharge or replace the batteries. This difficulty leads us to deduce that the life of a sensor is essentially dependent on the life of the battery. Therefore, energy-consumption management is a major constraint in this type of network.
- *Transmission scope.* This is an important criterion for the deployment of a WSN. It is limited by the radiation capacity of the antennas used and the signal strength involved. For example, the communication between two sensor nodes can only take place if the distance between them is not too great, i.e., a few tens of meters in practice. The longer the distance is, the higher the energy cost. This is especially true if there are physical obstacles between the two nodes that may prevent communication.
- *Storage and processing power.* An intrinsic characteristic of these sensors is their low storage capacity. Although they also have a processor, the sensors cannot perform very large operations due to their relatively low processing power. For example, “mote”-type sensor nodes are composed of an 8-bit 4 MHz microcontroller, 40 KB of memory, and a radio with a bit rate of about 10 Kbps. This remains true even for midrange nodes, such as “UCLA/ROCKWELL’S WINS”, which have a strong ARM 1100 processor with 1 MB flash memory, 128 KB of RAM, and a 100 Kbps radio.

#### 2.5.2. WSN Features

In addition to the sensor nodes’ intrinsic characteristics, there are other characteristics that are related to the network: sensor self-configuration, scalability, fault tolerance, communication capacity, network topology, bandwidth, and density.

- *Sensor autoconfiguration.* In a WSN, sensors are deployed either in a random manner by a missile, drone, or airplane, or manually deployed by a human or a robot, inside or around an observed phenomenon such as a field of war, volcanic surface, or sick patient [46]. Thus, each sensor node has the ability, on the one hand, to self-configure in the network, and, on the other hand, to collaborate with other nodes in order to dynamically reconfigure the network in case of network-topology changes [41]. As a reminder, each sensor in a WSN has a transmitting/receiving unit that allows it to communicate with the sensors that are close to it. By exchanging information with them, the sensor can then discover its neighbors and thus know the routing method that it will adopt according to the needs of the application. Autoconfiguration appears to be a necessary feature in the case of WSNs since, on the one hand, their deployment is done randomly in the majority of applications, and, on the other hand, the number of sensors is very high.
- *Scalability.* Unlike traditional wireless networks (personal, local, or extended), a WSN consists of a very large number of sensors (hundreds or thousands) [33]. The sensor network is scalable in that it has the ability to accept a large number of sensors that work together to achieve a common goal.
- *Fault tolerance.* In case of a sensor malfunction (lack of energy, interference with observation environment) or if new sensors are added to the network, the existing sensor nodes in the WSN must continue to normally operate without interruption [33]. This explains the fact that a WSN does not adopt a fixed but rather dynamic topology.
- *Communication ability.* It can take one of two aspects, multihop or one-hop. Multihop communication is less energy-intensive, and it remains the most sought-after type of communication for WSN applications that require low energy consumption. Finally, it should be noted that WSNs use a nonstandardized protocol stack. However, the majority of scientific work that deals with the WSN issue is based on the protocol stack proposed in 2002 by Akyildiz et al. [33], which is presented in Section 2.6.

### 2.6. Protocol Architecture

**Figure 6:**
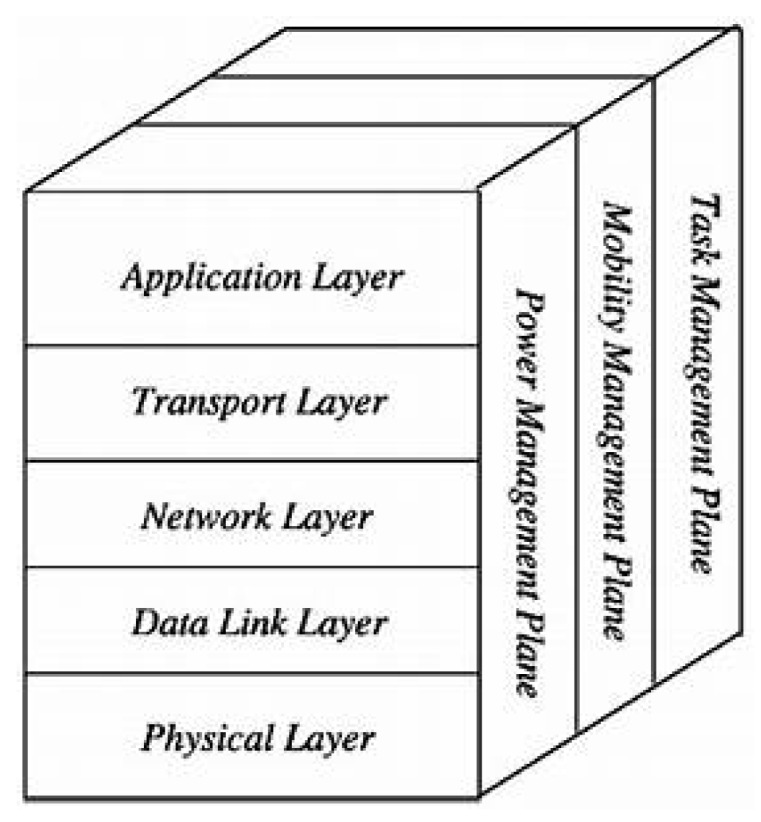
WSN protocol stack [33].

The role of the protocol architecture given in Figure 6 is to standardize communication between network components so that different vendors can develop compatible products (software or hardware). This protocol stack combines energy routing and management, and integrates the data with the network. It communicates efficiently (in terms of energy) through the wireless medium and promotes co-operative efforts among sensor nodes [45]. Depending on detection tasks, different types of application software can be designed and used in the transport layer. A WSN can be seen as a series of connections between sensors that enable them to communicate. The content, scope, size, speed, and connectivity of the network depend on the set of protocols and their implementation. This includes application, transport, network, link, and physical layers, and energy-, mobility-, and task-management plans.

- *Application layer.* Depending on the functionality of the sensors, different applications can be used and built on this layer.
- *Transport layer.* It is used to maintain the flow of data when needed in the applications used, especially when connected to the Internet.
- *Network layer.* This deals with the routing of data provided by the transport layer.
- *Data-link layer.* As the sensor network environment is noisy and nodes can be mobile, the MAC layer must ensure low power consumption in the data broadcast of neighboring nodes.
- *Physical layer*. This deals with data-transmission, -reception, -modulation, and -encryption techniques.
- *Energy-, mobility-, and task-management levels* are responsible for controlling the consumed energy, node movements, and task distribution across the entire protocol stacks. These levels allow sensors to co-ordinate their tasks and minimize energy consumption.

### 2.7. Dedicated Applications

**Figure 7:**
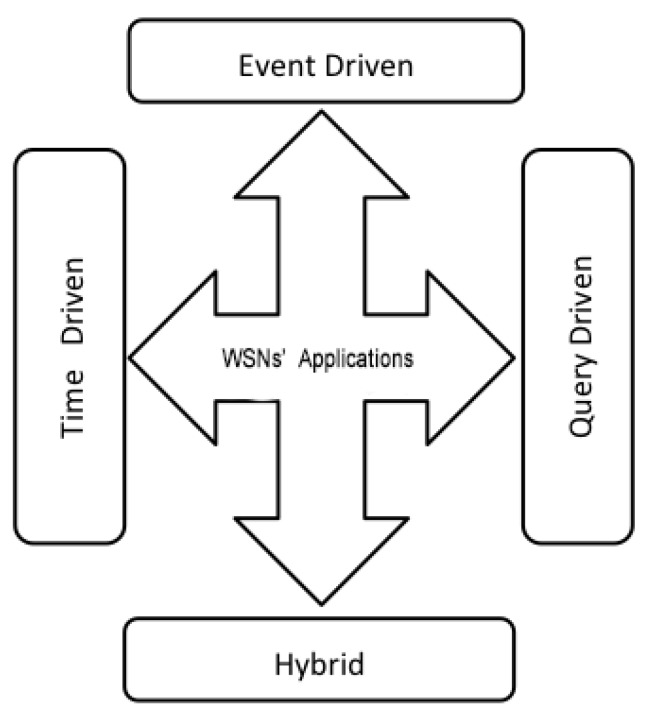
Dedicated WSN applications.

WSNs are in use in many areas, and most often with a varied architectural design. Moreover, sensor networks correspond to specific types of applications, as can be seen in Figure 7. A 2012 study by IDTechEx has reported that the worldwide market for sensor networks is growing, and is expected to grow from 0.45 billion to 2 billion between 2012 and 2022. We distinguish four types of WSN applications [32]: event-driven applications, periodic data-collection applications (also called time-driven applications), recommended execution applications or query-oriented applications, and hybrid applications.

#### 2.7.1. Event-Driven

**Figure 8:**
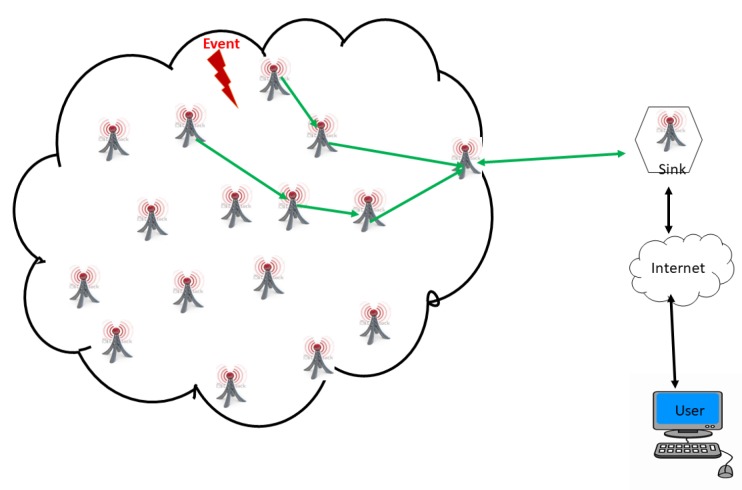
Event-driven applications.

This type of application is the most representative of WSN applications (see Figure 8). As an example, we can mention the detection of a forest fire or an earthquake, or the monitoring of the air quality in a given region. In this class of applications, sensor nodes must be able to detect an event that could happen at any time in any location in the area of interest. Thus, the sensor nodes must continuously collect this information and immediately react to abrupt changes in the sensed values. For this application category, no periodic control is required by the sink, and the data processing by the sensor nodes is simple: each node must compare the measured data with a fixed critical threshold. As soon as this value exceeds the threshold, the sensor node sends the data to the sink. In order to provide better performance, these applications must meet certain criteria, such as:

- A high density of sensor nodes is required to ensure high probability of event detection. This implies that the network must have very good coverage.
- Good coverage of sensor nodes that usually depends on the type of event to be detected.
- An exact location of all sensor nodes in the network is required. This is to locate the event through the sensor node in which the event occurs. Note that distributed location algorithms could be used for this purpose.
- Good network connectivity linked to transmission coverage so that communication protocols allow the alarm sent by sensor nodes to arrive at the sink with high probability and in the shortest time.

#### 2.7.2. Query-Driven

**Figure 9:**
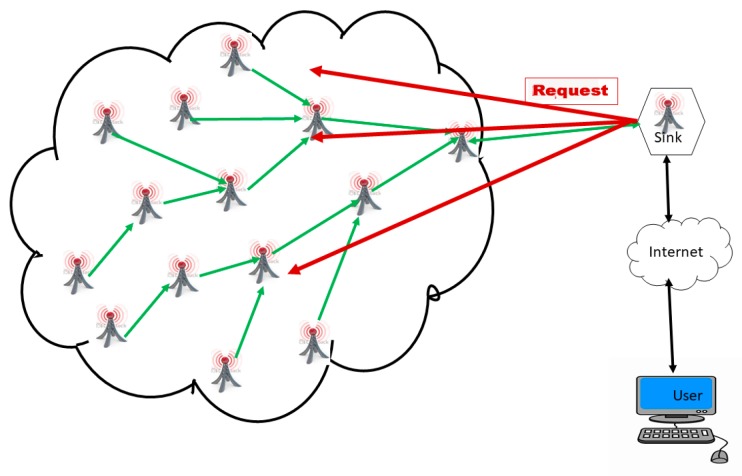
Query-driven applications.

In query-oriented applications (see Figure 9), the deployed sensor network tracks information only at the explicit request of a base station. This category of applications is intended for the specific needs of the user. The latter may seek, for example, to request information specific to a certain region, or to interrogate the sensors to acquire measures of interest. Generally In this case, the topological knowledge of the network and the geographical location of the sensors are necessary.

#### 2.7.3. Time-Driven

In this category, applications aim to periodically evaluate the measurements of the given physical quantities. Note, for example, the application of WSNs in the monitoring of the atmospheric pressure of a large area or variations in ground temperature at a small volcanic site [47]. These applications react in a given period sink. The sensors take a sample of the environment and transmit it by means of an appropriate communication protocol to the final sink. For the smooth operation of these applications, with better quality of service, it is important: that the sampling frequency is sink-chosen, that the connectivity of the network is kept under control, and to have communication protocols adapted to that kind of data. The sensor nodes used in this type of application can sleep, i.e., turn off their radio during periods of inactivity in order to manage the energy consumption of the network.

#### 2.7.4. Hybrid

This category of applications group the resulting applications of the two previous categories. Examples are home-automation applications, where we can evaluate the temperature of a room (event-oriented), or/and monitor a newborn (time-driven).

### 2.8. WSN Application Areas

**Figure 10:**
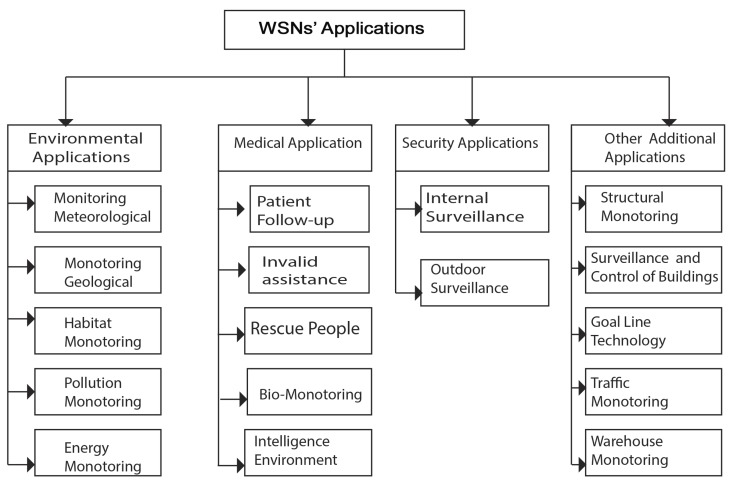
WSN applications.

The increasingly smaller size of the sensors, the ever-lower cost, the wide range of available sensor types (thermal, optical, vibration, etc.), as well as wireless-communication support, have widely spread the use of solutions based on WSNs [33]. Needs in terms of WSNs’ applications are growing every day. In 2006, the works of Ekici et al. [48] broke down sensor-network applications into four main areas, as described in Figure 10: environmental, health, security, and other applications. Furthermore, existing applications are extended in order to facilitate the design of other systems, such as the control and automation of industrial production lines.

#### 2.8.1. Environmental Applications

Nowadays, environmental problems, such as floods, fires, and pollution, are the most recurrent. The use of WSNs in environmental applications is becoming increasingly important and common, thus improving environmental awareness and the effectiveness of control methods [22,47,49]. Therefore, WSNs can contribute to the development of risk-response systems, natural-disaster detection, energy management, and other systems. Environmental applications can be broken down into five classes, as shown in Figure 10: weather monitoring, geological monitoring, habitat monitoring, pollution monitoring, and energy monitoring.

#### 2.8.2. Medical Applications

The health sector is increasingly using WSNs for healthcare needs, such as medical treatment, pre- and posthospital patient surveillance, and rescue operations [32]. In addition, sensor networks can help in solving important social problems, such as caring for the chronically ill and people with intellectual and physical disabilities. The field of healthcare includes patient follow-ups, invalid assistance, life-saving, biosurveillance, and intelligent environments. Furthermore, WSNs are used for monitoring physiological measurements [33]. They can also be used, for example, to monitor the vital functions of the elderly or people with health problems in order to alert medical devices. Similarly, we can analyze different physical measurements collected from athletes, the possibilities offered by a WSN to lighten data-collection devices.

#### 2.8.3. Security Applications

Some institutions can be monitored in real time by WSNs to prevent possible theft or fire. Another example is the application of sensor networks in intelligent transportation systems for monitoring roads or railways to prevent accidents [50]. In the military domain, these networks are used for surveillance inside and outside the theater of operations and in espionage. In the security field, sensor networks can significantly contribute to the reduction of financial expenses dedicated to the security of locations and human beings. Thus, the integration of sensors in large structures, such as bridges or buildings, could help detect cracks and alterations in the structure following an earthquake or the aging of the structure. Deploying a motion-sensor network can be an alarm system that can detect intrusions into a surveillance zone [41].

#### 2.8.4. Other Applications

Other applications of WSNs include [51,52] structural monitoring, construction monitoring and control, automobile surveillance, traffic monitoring, control of industrial processes, and warehouse surveillance. Usually, WSNs are particularly suitable for monitoring critical areas, thanks in particular to their autonomy and smaller size. In addition, possible applications include flood prevention, forest-fire detection, and the monitoring of nuclear facilities [53]. This type of network opens up new opportunities for managing the many problems that our present and future cities face, like traffic congestion, and energy and waste management.

Moreover, WSNs provide elements of answers to issues that remain relevant. They help find solutions for traffic observation and the management, especially the management of the traffic lights, the regulation of public street lamps, or the monitoring and management of centralized waste collection. Furthermore, in every-day life, home-automation and smart-building applications are particularly popular [45]. WSNs in these contexts provide innovative solutions for comfort, energy savings, and security [54]. For these applications, ZigBee alliance technologies offer a complete wireless protocol stack for different types of applications.

### 2.9. Challenges of WSN Design and Deployment

In most cases, the deployment of a WSN is closely related to the nature of the intended application. There is no generic model for these applications. However, several factors influence WSN design and deployment. The design of the WSN must find a compromise solution for the following challenges:

- *Energy.* A sensor node, by its size, is limited in energy resources, storage capacity, and processing. One of the most serious concerns is the lifespan of the sensor node, which is entirely determined by the amount of energy available and the rate of energy consumption (that is, the average energy consumption). Since improving the energy capacity of small batteries still requires a lot of effort, the goal of a long service life is difficult to achieve by increasing the amount of available energy. As a result, the design of energy-efficient MAC protocols, communication strategies, operating systems, and energy-efficient routing mechanisms provides effective ways to extend the life of the node [36]. The use of energy-recovery technologies for sensor-node power supplies has also been an alternative in recent years.
- *Quality of Service (QoS).* Quality is defined by the ability to interpret the information collected by the sink. In spite of the fact that the QoS requirements vary according to the different WSN applications, the two main measures of QoS are data reliability and latency [33]. Usually, a successful data-exchange rate between the sensor nodes and the sink must be above a certain threshold to ensure network reliability and functionality. Reliability can be further maximized, but this could be at the cost of increased energy consumption. Therefore, a compromise is necessary. In case of latency, for certain types of applications, the reception of the data detected on the sink is strictly timed, so that latency data can cause delay problems and lead to bad decisions, particularly in industrial-surveillance applications.
- *Security and privacy.* WSNs find their applications in various areas of life. Some applications manipulate private data. Moreover, WSNs are networks whose topology is very frequently subject to change. The deployment of sensor nodes is sometimes done in accessible areas; they may encounter technical interference or human intrusion, which can lead to confidentiality issues. Thus, the network should allow the detection of intrusions. It is therefore necessary to design robust and lightweight algorithms for data encryption, authentication mechanisms for privacy protection, and secure routing for data relays to protect the entire network against passive and active attacks, and denials of external service providers [55,56,57].
- *Adaptability.* The design of a WSN must be sufficiently flexible and adaptable to be deployable across a wide range of application scenarios. The entire network should be kept functional regardless if the number of sensor nodes is hundreds, thousands, or very small. Due to the mobility of sensor nodes and observed events, as well as the possibility that a sensor node can fail in the network, the overall topology of the network may be subject to frequent change. Therefore, the design of a WSN must be sufficiently intelligent and robust to deal with these dynamic topology scenarios [10,31].
- *Localization.* WSNs play an important role in the problem of locating elements in a given deployment area. Certainly, GPS is a well-known tracking system and the most used location system. However, it is not satisfactory for this use, because it accumulates handicaps. It is only available outdoors and if no obstacle obstructs the field of view of the receivers. In addition, an operation under dense foliage or in cities with narrow streets is not possible, which are very bad conditions. Moreover, it is particularly expensive, both in terms of hardware, multiplied to many copies in a network with high sensor density. Moreover, signal reception is very energy-greedy, which is not compatible with management problems of battery service life. Location is done in two steps: distance estimation to the other nodes, and triangulation. The development of new localization techniques has become a major concern for WSNs [33,58].

## 3. Overview of Big Data

This section aims at presenting an overview of the Big Data concept by presenting its categorization through the three-V model actually extended to five Vs, with an emphasis on different generation sources of these data. Moreover, a number of technologies on Big Data have been proposed in both industry and research applications. In view of its characteristics, the problem of Big Data is different from that of business intelligence, so Big Data challenges in the process of extracting knowledge from data are studied. These challenges are grouped into three phases: storage, processing, and exploitation of results. Therefore, using LS-WSN to collect, process, and exploit knowledge extracted from Big Data is an emerging trend.

However, we must say that the term Big Data is still an abstract concept. Sometimes called massive data or metadata, Big Data is an increasingly heterogeneous dataset, which is growing in volume such that it is difficult to process these data with conventional database-management tools [11,13]. In this context of data deluge, McKinsey, in its June 2011 publication “Big data: the next frontier for innovation, competition, and productivity” advocates the new era of Big Data [59]. Unfortunately, Big Data appeared much earlier, more than a decade ago, without being named, with the emergence of large data that cannot be processed using traditional techniques [29]. Large IT companies, such as Yahoo and Google, were the first to offer their own Big Data solutions. They were confronted very early with issues related to the storage and processing of large volumes of data. Therefore, Big Data is a new generation of solutions for collection, storage, real-time processing, and data analysis.

### 3.1. Characterization of Big Data

Characterization of Big Data is generally done according to the three Vs: *Volume*, *Variety*, and *Velocity*. Other complementary Vs are added, such as *Value* and *Veracity*.

#### 3.1.1. Volume

*Volume* is certainly the one best described by the term *Big* in “Big Data”. Volume refers to the amount of too-large information to be acquired, stored, processed, analyzed, and distributed by standard tools. This data volume is constantly growing. With the exponential growth in the number of electronic smart devices, the world is steadily becoming more connected. Moreover, connected objects will continue to grow over the coming years. For instance, according to computer company IBM, 2.5 exabytes, i.e., 2.5 billion gigabytes, of data were generated every day in 2012. This amount of data will reach 500 zettabytes by the end of 2019 [1]. Furthermore, some analyses report that, in the last two years, about ninety percent of these data has been generated. In most cases, most of the generated data come from the digital world. In 2013, IBM reported that about 12 terrabytes of tweets were created each day, and 5.2 billion daily Google searches in 2017. Table 1 highlights the volume of data generated by the digital sector. Volume is certainly the main criterion that comes to mind when we talk about Big Data. However, summarizing Big Data volume is too simplistic. There are other criteria, such as velocity and variety, to characterize Big Data.

#### 3.1.2. Velocity

*Velocity* refers to the dynamic and/or temporal aspect of the data, and their update and analysis time. Data are no longer processed and analyzed offline, but in real time or near-real time. They are produced in continuous streams, during which real-time decisions can be made. These are the data especially from sensors, requiring fast processing for a real-time reaction. In the case of such high-velocity data generating very large volumes, it is no longer possible to store them as they are, but only to analyze them in streaming, or even to summarize them; for instance, in time-sensitive processes like detecting frauds, Big Data must be used as they stream into industries in order to maximize their value. The challenge of Big Data applications is to recover data almost instantly, process them in real time, and extract useful information.

#### 3.1.3. Variety

*Variety* refers to the heterogeneity of formats, types, and quality of information. It is linked to the fact that these structured and unstructured data can present complex shapes because they have their origins in various and varied sensors (e.g., temperature, wind speed, hydrometry, humidity, and brightness), in exchanged messages (e-mails, social media, and exchange of images, videos, and music), in texts, online publications (e.g., digital libraries, websites, and blogs), recordings of purchase transactions, planning of scanned books, directories, log files, click streams, and information from mobile phones. Most of these data consist of public or private data, local or remote, shared or confidential, and complete or incomplete. When analyzing these data types together, new insights are found.

The three Vs are the major features of Big Data. A more relevant definition ofBbig Data could then be: data that are too large, too fast, or too broad in variety to directly store in traditional databases or processed by current algorithms [60]. The recent literature adds other complementary *Vs*, turning Big Data into Smart Data.

### 3.2. Toward Smart Data

Recently, a new term has emerged that tends to replace Big Data. This term is Smart Data. Why smart? Because not all data in Big Data are good to recover. Smart Data add a *V* to Big data, the *Veracity* of the data, which is directly related to value. Without Veracity and without quality data, *Value* is illusory.

#### 3.2.1. Veracity

Completeness *Veracity* or *Validity* refers to the quality of the data and/or the ethical issues related to their use. Not all data are intended to be analyzed. We must unload unusable data, called bad data, of poor quality for several reasons. This understands the problems of outliers or missing values (these problems can be solved by the volume of data), but also to the confidence that one can have in the data. If there are criteria for qualifying the quality of data, in the case of Big Data this quality check is made difficult or impossible because of the volume, variety, and velocity specific to Big Data [61].

#### 3.2.2. Value

The complementary *V* character that is *Value* refers to the potentiality of the data, especially in economic terms. It is thus associated with the use that can be made of these Big Data and their analysis, especially from an economic point of view. In this light, a recent study by Deloitte [62] states that *Viability* is named as the fifth *V*, and the sum of the five Vs is likened to *Value*. Value is the criterion resulting from the exploitation of Big Data; how to validate useful, reliable and accurate data from many large online datasets? Once done, how to extract value? In addition, Big Data is an interdisciplinary issue requiring cooperation from academia, industry, and business [63,64].

### 3.3. Big Data Generation

**Figure 11:**
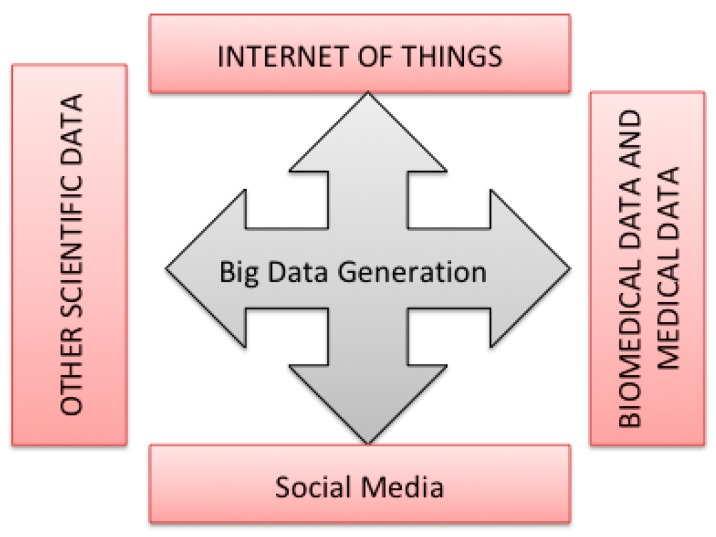
Taxonomy of Big Data sources.

In this section, we explore the main sources of massive data generation. As highlighted in Figure 11, we have four main sources of Big Data.

#### 3.3.1. Medical and Biomedical Data

The medical field with related services, such as bioinformatics, computer clinics, and other computer- and health-related activities, generate multidimensional data, and are a source of Big Data generation [65]. Examples are genome-related clinical projects and real-time health-monitoring systems using wireless sensors. These different techniques generate a lot of data. In addition to this, medical-imaging software produce large amounts of data, even with more complex features. In the effort to modernize medical trays, the Swiss government carried out different types of projects going toward this direction, so a project named ProteomicsDB was launched to handle genes with a size of 5.17 terabytes [66].

#### 3.3.2. Internet of Things

The IoT covers the connection of objects to the Internet through sensors to retrieve and exploit data from the physical world. The constant evolution of Internet technologies, coupled with the continuous decline in the cost of electronics, has today made it possible to deploy such systems on a large scale. The best definition of the IoT would be: an open and comprehensive network of smart objects that have the ability to self-organize, share information, data, and resources, and react to and act on situations and changes in the environment [67]. The IoT is therefore seen as the next online revolution [60].

#### 3.3.3. Social Media

Social media are one of the main sources of massive data generation. Nowadays, social media platforms, such as Facebook, Youtube, Twitter, and Instagram, produce large amounts of data. For instance, SocialBlade.com reported that Instagram, with 7.1 million active users, has more than 34.7 billion shares of active photos, and more than 3.25 billion hours of videos are watched in one month. In the same light, Twitter has 355 million active users every month, averaging 665 million tweets per day. More than 5 billion people worldwide call, text, tweet, and browse on mobile devices [60]. The number of email accounts created around the world increased from 3.3 billion in 2012 to more than 4.3 billion by the end of 2016 at an average annual rate of 6% over the four years. In 2012, a total of 89 billion emails were sent and received daily, and this value is expected to increase. An average annual rate of 13% over the next four years will exceed 143 billion by the end of 2019 [68].

#### 3.3.4. Scientific Data

Scientific data are a particular source of generation of Big Data. In most cases, scientific projects carried out in areas such as astronomy, earth sciences, and projects related to the study of the ocean and its reconstruction via imagery, are projects generating large quantities of data. As an example of the data generated by different scientific projects, 2MASS (both Micron All-Sky Surveys) generates 10 terabytes, DPOSS (Le Palomar Digital Sky Survey) generates 3 terabytes, GBT (Green Bank Telescope) produces 20 petabytes, GALEX (The Galaxy Evolution Explorer) has 30 terabytes, LSST (Large Synoptic Survey Telescope) has more than 200 petabytes expected, SDSS (Sloan Digital Sky Survey) produces 40 terabytes, SkyMapper Southern Sky Survey has 500 terabytes, Pan STARRS (Panoramic Survey Telescope and Rapid Response System) has more then 40 petabytes planned, and SKA (Square Kilometer Array) has more than 4.6 exabytes expected [68].

### 3.4. Areas of Utility of Big Data

**Figure 12:**
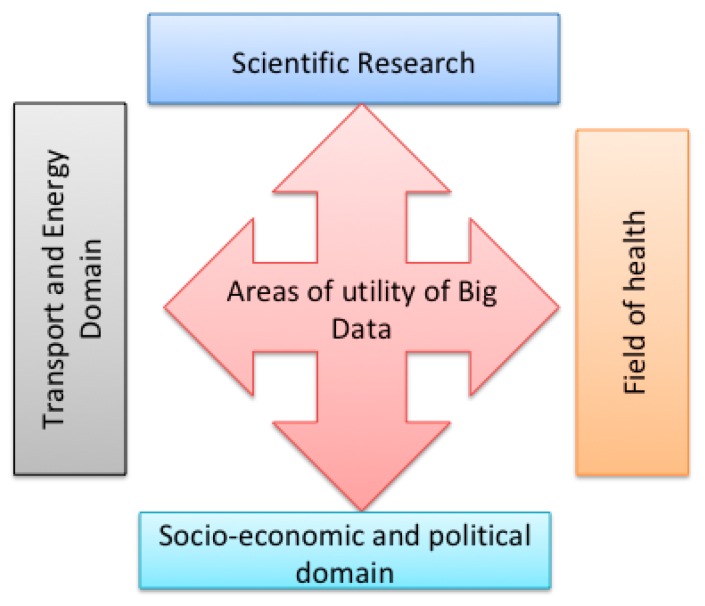
Taxonomy of Big Data utility domains.

Big Data present application perspectives in all activity sectors, scientific, technical, and socioeconomic, from data recovered from the operation of aircraft engines to better maintain or design them, up to data for specifying our relationships on social networks that can be used by banks to estimate the quality of our credit [69]. Figure 12 presents a taxonomy of Big Data utility domains.

#### 3.4.1. Scientific Research

In the scientific and technical fields, scientists and engineers particularly face Big Data generated automatically by sensors or measuring instruments. In medical research, technologies associated with Big Data have led to spectacular advances in the analysis of the human genome. This knowledge of the genome, coupled with others, makes it possible to better understand the evolution of pathologies, and to improve preventive measures and even care protocols.

#### 3.4.2. Health

In the area of health, McKinsey estimates that Big Data could save the U.S. healthcare system USD300–450 billion in the Big Data revolution in healthcare reports. These savings include prevention, with a follow-up of patients to change their habits, diagnosis, helping doctors to choose the most appropriate treatment, medical staff, determining if a patient needs a nurse, a general practitioner, or a specialist, cost control both by automating the reimbursement procedures and by detecting fraud, and finally innovation through multiple contributions of intensive computing to the understanding of living and the improvement of treatments. Similarly, thanks to Big Data, it is possible to better prevent certain diseases or epidemics, or improve the treatment of patients.

#### 3.4.3. Socioeconomic and Political Domain

In the socioeconomic field, Big Data in general can be used to simplify or adapt offered services by better listening to users and understanding how they use these services [70]. For instance, Google Analytics offers companies, as well as public administrations, the opportunity to improve their website design by analyzing Internet user visits. Moreover, in education, with distance education such as the Massive Open Online Course (MOOC), the Big Data treatment is used to analyze student activities, including spent time, how to follow the programs, stop and return in pedagogical videos, and parallel Internet searches, to improve teaching methods.

#### 3.4.4. Transport and Energy Domain

In the field of transport, population movements can be modeled to adapt infrastructure and services such as train schedules. For this purpose, data from transit passengers, bicycles, and shared cars, as well as from the geolocation of people or cars, are used, such as cellular data and satellite tracking systems. In the field of energy and sustainable development, smart metering systems (electricity, gas, water) generate Big Data that can rationalize energy consumption [71]. In addition to offering citizens the opportunity to better control their consumption, these meters allow remotely cutting, with the agreement of customers, the supply of equipment to avoid overloading the network. In air transport, by combining sensor data on airplanes with weather data, air corridors can be modified to save fuel, improve aircraft design, maintenance, or safety [72].

### 3.5. Big Data Challenges

**Figure 13:**
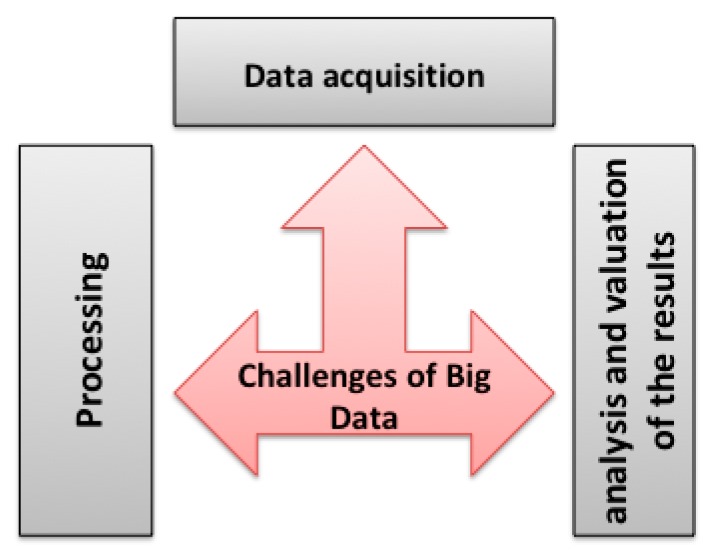
Big Data challenges.

This section presents the challenges to overcome in order to take advantage of the exciting opportunities offered by the advent of Big Data in various business sectors. The different challenges of exploiting Big Data (see Figure 13) are categorized according to the three phases of the process of extracting knowledge from data: data acquisition, processing, and exploitation of results.

#### 3.5.1. Data Acquisition and Storage

Data acquisition refers to data acquisition, storage, and preprocessing. Big Data are produced continuously, and volume is growing at an exponential rate. At this speed, the acquisition of these data is a real challenge for researchers and industrialists. Indeed, the nature of Big Data already presents several challenges [73]:

- *Heterogeneity.* In general, data-mining algorithms and machine learning expect homogeneous data, and cannot understand the fine distinction between them. IT systems work the fastest if they can store multiple items that are the same size and structure. Thus, Big Data must be carefully structured as a first step in their acquisition process [74].
- *Scale.* The first thing everyone thinks about Big Data is size. The management of large volumes of data and high growth has been a major problem for many decades. The volume of data is increasing faster than IT resources. Unfortunately, parallel data-processing techniques that were useful in the past for processing data between nodes do not apply directly to intranode parallelism, since the architecture is very different.
- *Delay.* The larger the dataset to be processed is, the longer it takes to analyze it. Designing a system that effectively treats size is also likely to result in a system capable to more quickly process a given dataset size. However, it is not only this speed that is generally heard when we talk about Velocity in the context of Big Data [75]. There are many situations in which the result of the analysis is immediately required. Given a broad dataset, it is often essential to find elements that meet a specific criterion. The analysis of all the data to find the appropriate elements is obviously impracticable [76].
- *Privacy and Security.* One of the key value propositions of Big Data is access to data from multiple and diverse domains. Security and privacy play a very important role in Big Data research and technology. In areas such as social media and health information, more data are collected on individuals. It is therefore feared that some organizations will know too much about individuals, developing algorithms that randomize personal data into a large dataset problem [77].

In terms of data storage, relational databases remain the reference, but these systems find their limit against Big Data. These widely used tools guarantee the maintenance of ACID (Atomicity, Coherence, Isolation, and Durability) properties. The management of large volumes of data, especially in the context of data warehouses, always faithful to the relational model, solutions like Teradata rely on the distribution of data on different disks, allowing a parallelization of query execution. However, these solutions do not allow massive data management beyond a certain volume. Thus, different new solutions have emerged. All of these solutions rely on the distributed (partitioned) storage of cluster data. Given that Brewer’s theorem is quite easily proven, no distributed system can ensure consistency, availability, and the possibility of being partitioned. The consequence is that, in these new storage solutions, it is not possible to provide the ACID properties, and a loosening of these properties is necessary.

#### 3.5.2. Data Processing

This treatment refers to all techniques and methods dedicated to the exploitation of Big Data. The data must be presented in a suitable format in order to be used as input to generic data-processing algorithms. The challenge of Big Data processing is to be able to propose methods and techniques to manage the complex nature of these data (Velocity, Volume, and Variety) [68], and, in most cases, the treatment process. Massive data are performed in a distributed environment. Traditional data-processing methods are not suited to the context of Big Data. The design of new methods or the adaptation of existing methods for the processing of these data in the context of a distributed environment remains a topical research issue.

However, the use of distributed data-analysis algorithms, including search, learning, decision support, and visualization, requires the rewriting of existing algorithms or the adaptation of Big Data to the data-input format of existing algorithms in a distributed context, but also to use specific hardware environments to run them in distributed mode, for example, multicore and many-core machines, or computing grids. The current limits to the distribution of Big Data analysis algorithms, analysis is taken here in a broad sense, including search, learning, decision support, and visualization, resides as much in algorithmic difficulty as in the hardware architecture of execution. Thus, most search algorithms are not easily distributed, and not necessarily with a MapReduce approach, and it is sometimes necessary to find new techniques to achieve efficient parallelization [78].

Although sensors are limited in terms of resources due to the fact that they are equipped with an intelligence allowing them to collaborate to carry out a task, sensor networks can be an interesting means for capturing and managing Big Data.

## 4. Big Data Collection in LS-WSNs

**Figure 14:**
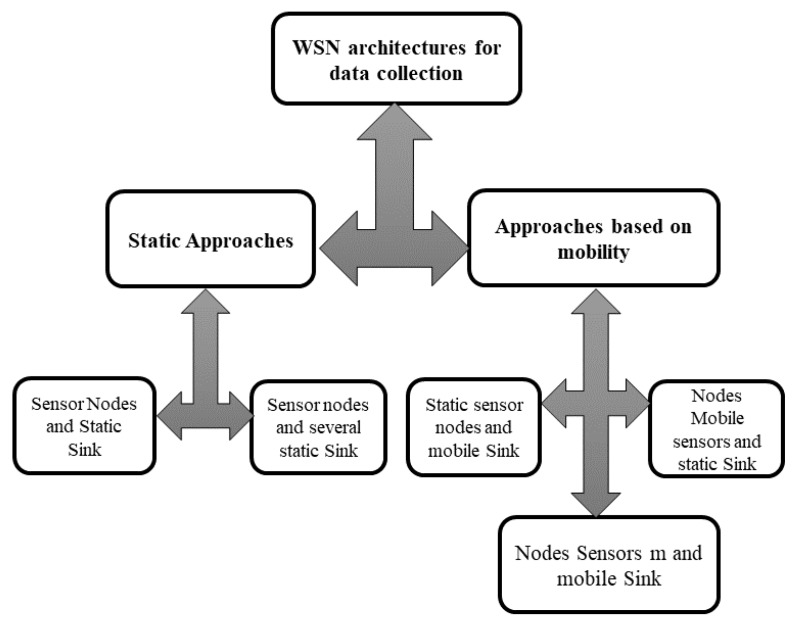
Different architectures of WSNs.

The collection of data by a WSN is based on a well-defined architecture. The real problem lies in choosing an optimal architecture. In this section, we present the different architectures proposed in the literature in order to detect those that are compatible with large-scale WSNs. The work of References [25,32,33,41,45,79] proposed several network architectures dedicated to data collection. These architectures are built around two main approaches: one based on a static network, and the other on network mobility. Figure 14 presents the different data-collection architectures proposed in the literature.

### 4.1. Static Architectures

This is the classical approach encountered in the literature. It consists of deploying a set of sensors in an area of interest in order to gather information. In this architecture, all network components are static. On the other hand, the deployment of the network can be achieved in two ways: deterministic or random.

#### 4.1.1. Static-Sink and Static-Sensor Architecture

This architecture consists of a large number of sensors capable of collecting and transmitting environmental data in an autonomous way. The position of these sensor nodes is not necessarily predetermined. They can be randomly dispersed across the area of interest. This type of architecture is characterized by a high density in order to find at least one path between any two network nodes. Moreover, one of the major drawbacks of this architecture is that it shortens the lifetime of the network. The authors in References [25,80,81,82] reported a problem of rapid depletion of batteries from neighboring sensor nodes to the sink. The relay of data from the source sensor to the sink is the source of the network’s nonuniform energy consumption. Thus, neighboring sensor nodes at the sink, being the most requested in the data transfer to the sink, quickly exhaust their energies, and the sink, in turn, is out of order. This fact reduces the lifetime of the network. For instance, applications that use this type of architecture include the VOLCANO [47], GLACSWEB [83], and the Habitat Monitoring on GDI (Great Duck Island) projects [84].

#### 4.1.2. Architecture with Sensor Nodes and Several Static Sinks

**Figure 15:**
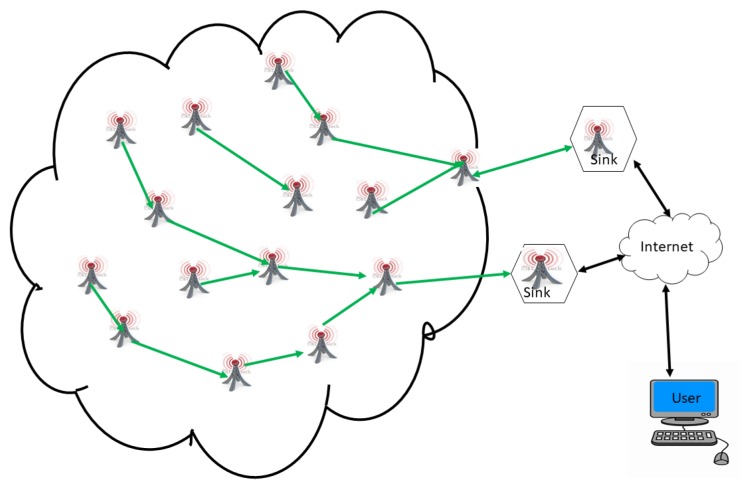
Architecture with sensor nodes and several static sinks.

Unlike the previous architecture, the architecture with sensor nodes and several static sinks (see Figure 15) consists of multiplying the number of sinks in the network in order to increase the lifetime of the network. However, the problem of battery depletion of the sensor nodes lying at a jump from the sinks remains. Sensor nodes of this architecture, the choice of one sink compared to another, remain a challenge in this kind of architecture [85].

### 4.2. Architecture Based on Mobility

**Figure 16:**
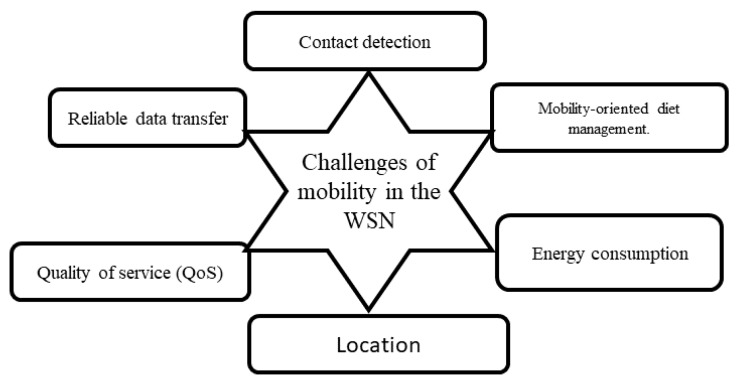
Challenges of mobility in a WSN.

In order to maximize network lifetime and reduce latency in WSNs, several works [48,86,87,88] have introduced mobility into WSNs. Mobility in WSNs is useful since it has a number of advantages that include good connectivity, reduced deployment cost, reliability, and energy efficiency [84,89,90,91]. Although mobility brings significant benefits in terms of increased network lifetime, it presents several challenges for sensor networks. These challenges (see Figure 16) are, among others, contact detection, mobility-oriented power management, reliable data transfer, QoS, location, and energy consumption [92,93,94].

Different kinds of mobility in WSNs have been proposed: static-sink architecture and moving sensor nodes, architectures with mobile and static-sink sensor nodes, architectures with mobile sinks and mobile sensor nodes, architectures with sensors, and multiple static-sink architectures with sensors and multiple mobile sink and hybrid architectures.

#### 4.2.1. Static Sink Architecture and Moving Sensor Nodes

This architecture has the particularity that the sensors are mobile. This approach has been the subject of much work in recent years [48,86,87,88]. By the fact that the sensors are mobile, this architecture considerably reduces the number of sensors to deploy. Communication in this architecture is direct. This architecture helps maintain good network coverage. Moreover, this architecture allows a balanced consumption of energy at the sensor level, and the amount of data collected by the sink is significant. For instance, these projects use this architecture in which sensor nodes move while the sink is fixed: the COW project [95] and the CENWITS project [96].

#### 4.2.2. Architecture with a Mobile Sink and Static Sensor Nodes

Unlike the previous architecture, all nodes are static and the sink is mobile here. Data collection is done once the sink reaches a given sensor node and that sensor detects the presence of the sink. This architecture poses the problem of detection of the sink by the sensor nodes. However, it represents an interesting solution, consisting of using mobile agents to collect data gathered by the sensors. Akyildiz et al. (2002) [33] as well as Jain et al. (2006) [42] reported that mobile agents called MULEs (Mobile Ubiquitous LAN Extensions), mobile sinks, or mobile data collectors move in a random or controlled way to collect data accumulated at the sensors [26,97]. Data collection is done with the approach of the mobile agents of the fixed sensor nodes. These mobile agents can be humans, robots, animals, or vehicles. This architecture has the advantage of relaxing the constraint of high densities in favor of better connectivity to the WSN deployed in an area of interest. Thus, such a WSN architecture has the advantage of avoiding collusion and loss of data induced by networks of wireless sensors with high density of nodes as in References [41,79].

#### 4.2.3. Architecture with a Mobile Sink and Mobile Sensor Nodes

In this architecture, mobility is considered at the level of the sensor nodes as well as at the level of the sinks. This architecture has the same advantages as those of the mobile sink and the static sensor node architectures. This architecture has been implemented in several scientific and industrial research projects. For instance, this architecture was implemented as part of a zebra tracking project in Kenya; the sensors were deployed on zebras from Sweetwaters Reserve to Mpala in central Kenya, and the sink was positioned on a vehicle. This project was one of the first studies carried out in the field of environmental monitoring that simultaneously took into account the mobility of nodes and sink. As a type of application using this type of architecture, we obviously mention this zebra tracking project (ZebraNet) [84].

#### 4.2.4. Architecture with Sensors and Multiple Static Sinks

This architecture differs from all architectures presented so far. It consists of using several sinks with a high density of sensor nodes. The data collected by the sensor nodes are transmitted to the end users via one of the network sinks. The big problem with this architecture lies in the difficulty of choosing the sink. One can also perceive the complexity of such a solution in Reference [81], which proposes a routing optimization solution in a multi-to-many architecture. This proposal also suffers from the clear problem of a lack of evolution. Indeed if the sensor network grows, so as not to fall back into the same problems as a single-sink architecture, we would have to add sinks, which cannot be done automatically. In addition, this solution still does not allow a direct connection between the end user and the sensor nodes.

#### 4.2.5. Architecture with Sensors and Multiple Mobile Sinks

This strategy is almost identical to the previous one, with the difference here being that the sinks are mobile. This solution consists of placing a plurality of mobile sinks in a single WSN [98,99]. An important problem with this solution is the difficulty of routing the data collected by the sensors. Juang et al. (2002) [84] tried to solve this by proposing a solution to optimize routing.

#### 4.2.6. Hybrid Architectures

There are also solutions with a multisink architecture. The lifetime of the WSN could be significant when using multiple mobile receivers instead of static receivers in data collection. Other works, with several mobile sinks, focused on reducing the energy consumed by sensors, or to maximize the lifetime of the global network. These proposals often come from a lack of scalability. Indeed, if the size of the sensor network increases, it is to avoid the problems of an architecture with a single sink, which can not be done automatically. Moreover, this solution does not allow a direct connection between the user and the sensor.

### 4.3. Comparison of Data-Collection Architectures in the Context of LS-WSNs

The different architectures analyzed in this section each have advantages and disadvantages, the main reason being that the design of these architectures is determined by the goals that we set. Otherwise, in the context of LS-WSNs, the density of sensors deployed in a vast field of interest is high. This makes us say that even if mobility brings more advantages than inconveniences to the sensor networks, if this mobility is on the side of the sensor nodes, it would reduce the number of nodes to deploy. We can therefore say that these kinds of architecture are not suitable for LS-WSNs. Regardless of what the sensor and static-sink networks consume in terms of energy, they are adapted as the network scales up. The real challenge remains the optimization of energy consumption in this network. For this, the different techniques used in the context of so-called *conventional* WSNs must be adapted to better respond to an environment with a high concentration of sensors. A comparative study of the different architectures is presented in Table 2.

## 5. Data Transferring in LS-WSNs

**Figure 17:**
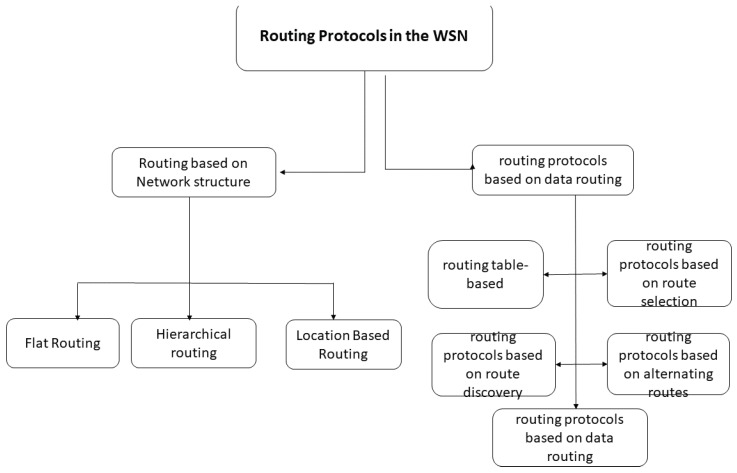
Taxonomy of routing protocols in WSNs.

The data captured by the nodes must be transferred to a collection point in the network called a sink. Routing is the process of finding a cost-effective route in terms of power consumption in order to transfer the data from the sensor nodes to the sink. The sink can be connected to a remote user via Wimax, LTE, a satellite, or another wide transmission system. The user can address requests to other nodes in the network, specifying the type of required data and harvesting them through the sink node. Routing in LS-WSNs remain a very active area of research, and several routing protocols have been proposed [12,36,100,103,105,106,107,108]. Recently, routing protocols for WSNs have been the subject of several studies, and various studies have been published. These protocols can be classified according to two main axes [45,79]: the structure of the network and the type of operation to be performed (see Figure 17).

### 5.1. Network Structure-Based Routing Protocols

Routing protocols are distributed according to network structure, which plays a very important role in the data-routing process. There are three main categories of protocols based on network structure. These protocols are:

- *Flat routing.* Called ‘data centric’ as well, all sensor nodes here have the same tasks to perform. This is the first approach used in the routing of data in the WSN. It is based on the collaboration of all the nodes of the network. Data properties are specified by a naming system by attribute (attribute, value) because of the difficulty of assigning a global identifier to each node given their large number. Among their advantages is their simplicity, hence the possibility of establishing communications at no additional cost where each node would only need information from its direct neighbors. The disadvantage is the depletion of the energy resources of the nodes close to the base station because all traffic toward the latter obligatorily passes by them.
- *Hierarchical routing.* This approach is based on the formation of clusters (common areas) [36,85,100]. The principle is to route the data collected by each node of the cluster to its head of area, the Cluster Head (CH), which, after processing their common parts, forwards them to the next destination (If the CH cannot directly reach the station, basic information is routed to the next zone leader). The advantage is the reduction of communication and energy costs by minimizing the number of messages circulating on the network, since CHs apply aggregate functions on the data of the cluster, which makes it possible to combine them. The disadvantage concerns the size of the network. In addition, as network size increases, the CH election process becomes critical and greedy in resources.
- *Location-Based Routing.* The identification of the geographic locations of the sensor nodes on the gathering area is of paramount importance for the data-routing mechanisms in the WSN. This location information allows the calculation of sensor positions and the distances between them to build the shortest paths between a source node and its destination. This routing approach is more energy-efficient because it dispenses sensor nodes from using random or probabilistic methods to search for routes [109,110]. In addition, the location of the nodes (and consequently of their regions) makes it possible to only broadcast requests to these regions and to avoid their diffusion in broadcast mode (global diffusion to all the nodes), thus significantly reducing the number of transmissions. The disadvantage is the need to equip sensor nodes with a satellite tracking system, such as GPS, which consumes a lot of energy.

### 5.2. Feature-Based Routing Protocols

In this section, routing protocols are categorized according to the features they implement. These features are based on data routing, route selection, route discovery, and alternating routes.

- *Routing protocols based on data routing.* In this kind of protocol, each sensor node must choose a node of its neighborhood to deliver it the task of conveying these data. This decision is made using different techniques, including table-based routing, broadcasting, and filtering by neighbors, or flooding.
- *Routing protocols based on route selection.* Route selection is the process of choosing the best route to a sink from the potential routes depending on the constraints of the network and the sensor nodes. Although most protocols only choose a better route (single-path routing), multipath protocols select and save more than one path for different purposes, such as energy, traffic balance, reliability, and robustness. The entities involved in the selection process may include the source sensor node, the intermediate nodes, or the sink node.
- *Routing protocols based on route discovery.* Each sensor node of a WSN acts as a router and participates in the routing of data to the sink. Many debates and works have discussed when and how many routes each node must maintain in its routing table. Indeed, a node must memorize the roads to reach a sink, or only search and keep track of roads when it has an interest in reaching the sink. As a result, three types of routing protocols are distinguished depending on how routes are maintained in the routing tables. Route discovery is categorized into proactive, reactive, and hybrid routing protocols.
- *Routing protocols based on alternating routes.* In a WSN, from a sensor node, a routing protocol can maintain a single path that allows it to reach a sink. It can also maintain multiple paths to reach a single destination. We distinguish two classes of protocols: single path and multiple paths.

### 5.3. Data-Collection Steps in LS-WSNs

**Figure 18:**
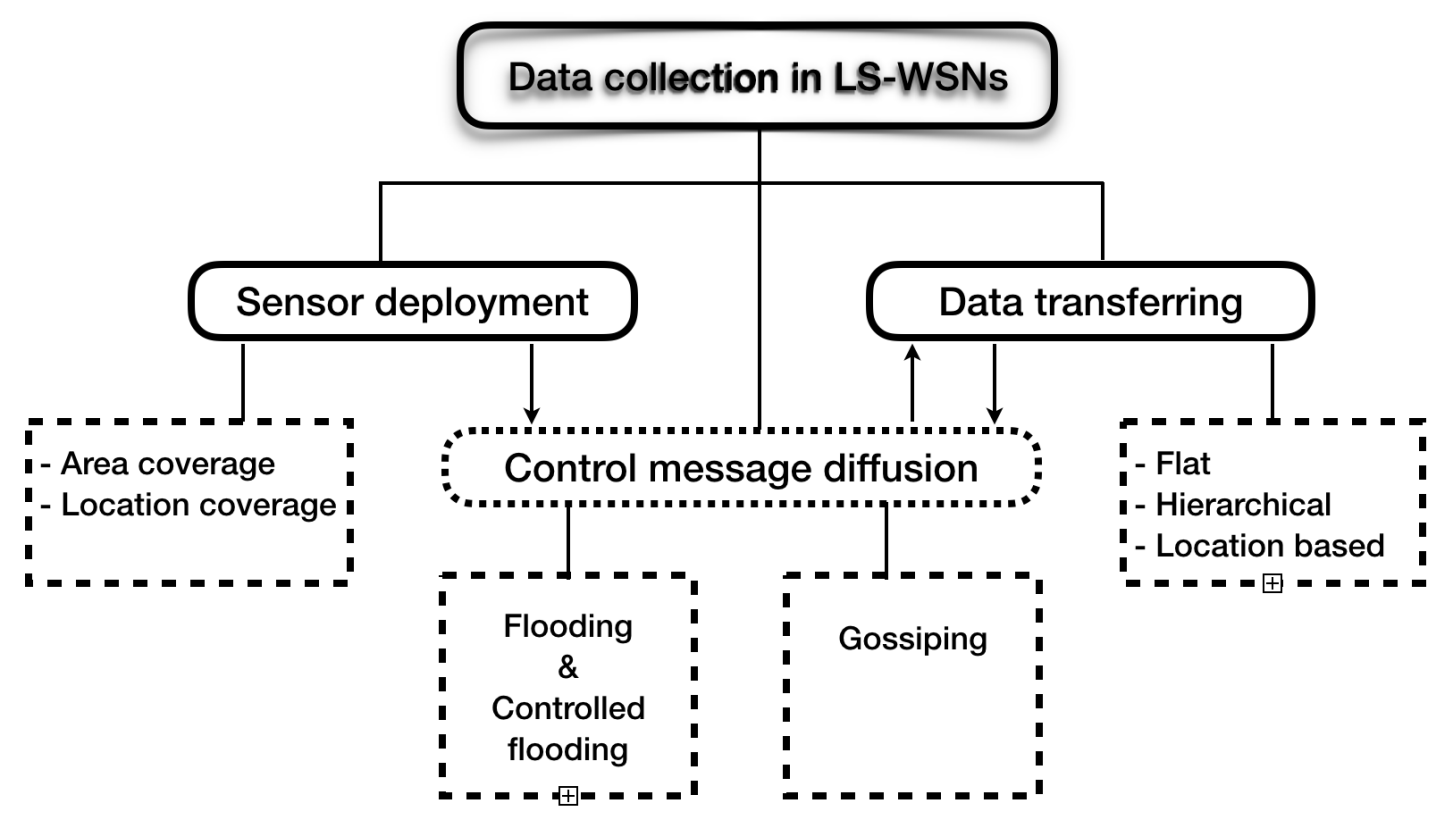
Flowchart of data collection in LS-WSNs.

The flowchart given in Figure 18 summarizes the major steps of data collection in LS-WSNs. According to application areas of sensor networks, the issue of deploying large-scale sensor networks for coverage purposes can be categorized into area-based coverage deployment that requires sensors to be deployed in the whole field, and location-based coverage deployment that requires sensors to be deployed in specific locations [111]. The diffusion of control messages is commonly done by flooding (and controlled-flooding) methods and gossiping methods [36]. Finally, the data-transferring step relies on knowledge recorded by the control message dissemination step to achieve data collection by adopting either flat-routing, hierarchical-routing, or location-based-routing schemes.

## 6. Challenges of Big Data Collection in LS-WSNs

With increasingly miniaturized and integrated sensors, a new paradigm of sensor networks is emerging in LS-WSNs. These networks are based on the cooperation of a set of nodes to perform various operations, such as monitoring and data collection, on the environment in which they are deployed. The term ‘large-scale’ refers to the fact that these are networks deployed over a large area with high sensor density. Consequently, all techniques and methods that can be used in so-called small WSNs cannot be directly used on these networks. They must undergo a modification to adapt to the extension of the network range. In general, a sensor is a resource-limited device whose energy resources and sensors are deployed in most cases in hard-to-reach areas, making it difficult or impossible to change the batteries. The management of energy resources is a strategic issue to increase the duration of a sensor network [24,46,108].

Even though WSNs have been used in a number of applications, data collection is the main activity of WSNs, whether LS-WSNs or not. For researchers, this operation consists of finding solutions to maximize data collection, and for others to minimize the energy consumption at the level of the sensors. In the literature [33,45], it appears that of all the basic functions of a sensor, communication is the most energy-consuming activity. This energy consumption largely depends on communication distance. Indeed, to reduce this consumption and extend the life of the sensors, and therefore that of the network, several techniques and methods have been developed in recent years. In most cases, these methods relax several constraints, such as latency, coverage, and QoS, as well as the Quality of Experience (QoE) of the network in favor of extending network lifetime.

In addition, the requirements specified by data-collection applications in LS-WSNs also lead to a number of issues that need to be considered. Among them, WSN coverage is an essential issue in sensor network deployment, which must make sure that sensors deployment in a given area of interest may need to allow an acceptable coverage, especially for certain WSNs’ applications. Moreover, in certain cases, sensors should be located in particular locations in order to more accurately gather data. This fact is important for industrial 3D sensor networks or sensors deployed in aircrafts [112,113]. Furthermore, the way and rate of acquiring data cannot be the same for different kinds of data, such as vibration, temperature, movement, and humidity [10,114,115]. These issues, if not handled well, lead to an unbalanced energy consumption resulting to power wastage and a short network lifetime. Otherwise, delivering data to the base station while minimizing the rate of data loss is also a challenge. Some works focus on network structuring and data aggregation [36,80,100,109,116,117], while others rely on routing techniques to reduce the energy consumption of sensor nodes [12,46,103,118,119]. However, operations like data aggregation, compression, and fusion remain difficult to apply in the case of large amounts of structured or unstructured data. It is, therefore, necessary to develop novel schemes for data collection in LS-WSNs.

Moreover, in terms of network structuring, hierarchical networks coupled with clustering techniques based on multihop communication seem to be the appropriate solution for LS-WSNs. These techniques reduce intranode transmission distance in the routing of the collected data. This is economical in terms of energy consumption compared to direct communication, that is to say, that each sensor node directly routes these data to the sink without a relay. In contrast, clustering results in higher latency than in flat structuring [120,121]. In addition, in this type of architecture, the most common problem is called the access point problem [122], that is to say, that sensor nodes that are in proximity to the sink are rapidly depleting their energy compared to other sensor nodes. This is justified by the high concentration of data traffic to the sink. Thus, network coverage rate is affected, with an increased risk of sink disconnection.

Furthermore, to overcome the disadvantages of multihop communication, other works propose to multiply the number of sinks within a network. Thus, a WSN can have several static sinks [120]. In this architecture, in general, these sinks have more hardware resources compared to other sensor nodes [94]. They are used to collect data from other sensor nodes, and to reduce the energy consumed in transmissions [120]. The data are then sent from the sinks to the end user via an Internet or satellite connection [94]. In this approach, the problem is the choice of a sink compared to others. Thus, in most jobs, metrics such as distance are used to determine which sink is closest to the sensor node to which the data will be sent. However, the issue of the optimal deployment of sinks remains a big problem, because it is difficult to choose the right place to deploy the sinks in order to balance the exchanges between nodes and sinks [102,122]. Even though static sinks are deployed in optimal locations, we still have the access point problem, which is that the nodes near the sinks consume more power. Sinks can be disconnected from the rest of the network if the surrounding nodes exhaust their battery. Thus, the optimal position of the sinks ensures good coverage of the area of interest, but not permanent coverage [93].

Another study [81] proposed an approach based on sink mobility. In addition to saving energy consumption at the sensor level of sink mobility, it also satisfactorily reduces increased data latency [15,24,40]. By choosing to organize the cluster network, this reduces the time spent in the sensor nodes and therefore increases the frequency of its visits to the cluster heads. In addition, using a method called service cycle can further reduce power consumption [102,122].

Furthermore, the problem of storing and computing very large amounts of collected data from LS-WSNs needs more attention. In this light, some companies, such as Nokia and Apple, integrate a number of sensors in their mobile phones and other smart devices on the Internet. This leads to the collection of petabytes of data each year. Certainly, some distributed database solutions, such as Google’s Bigtable, Amazon’s Dynamo, Windows’ Azure Storage, and Apache Hadoop, can achieve the storage of such large amounts of data. However, unless these Big Data are associated with programming models, such as Mapreduce, the existing distributed database solutions cannot process the large amount of data generated by sensors from LS-WSNs. Unfortunately, a number of works are focused on sensors’ interconnection and power-efficient WSNs [21,36,121]. In spite of the number of interesting challenges that should be addressed in sensor networks, large amounts of data collection, storage, and processing schemes in LS-WSNs should be developed.

## 7. Conclusions

The application prospects of WSNs are rich and varied. The use of these networks in the context of Big Data shows the ability of these networks to overcome inherent constraints to meet certain requirements. In Big Data, we expect a heterogeneous dataset that is continuously produced by the IoT to such an extent that it cannot be captured, managed, and processed by traditional systems. Such data, given their characteristics, are an interesting challenge for their acquisition and processing by LS-WSNs. In this paper, we conducted a comprehensive review that proposes a better approach to the collection of Big Data. Thus, in this context, to better address the issue of collecting Big Data, we first presented horizontal overviews of WSNs and Big Data. Then, we reviewed Big Data collection architectures, as well as data-transferring schemes. Moreover, the challenges of Big Data collection in LS-WSNs have been discussed. From the systematic analysis of standpoints of various authors regarding Big Data collection in LS-WSNs, we provided a detailed review of the open issues with their associated challenges in order to motivate and guide future researchers.

## Figures and Tables

**Table 1:** Volume of created data (Sources: bloomberg.com, IBM, BBC, onereach.com, Internet Live Stats, Internet World Stats, Forbes, Data Never Sleeps.).

Source	Production
YouTube	- Users upload 400 h of new videos each minute of every day.
	- Each month, more than 1 billion unique users have access to YouTube.
	- Users watch more than 4 million videos every minute.
	- Users watch 5.97 billion hours of videos each day.
Facebook	- Users click the Like button on more than 4 million posts every minute.
	- Every day, 5.75 billion likes are registered.
	- Every day, 4.3 billion messages are posted.
	- 100 terabytes of data are uploaded daily.
	- Over 2 billion monthly active users in 2017.
	- The site has been translated into 70 languages.
Twitter	- 656 million tweets every day.
	- The site has over 355 million of active users per month.
Instagram	- 7.1 million active users per month.
	- 34.7 billion shares of active photos per month.
	- 3.25 billion hours of video are watched in one month.
Foursquare	- This site is used by 45 million of people worldwide.
	- This site receives over 5 billion check-ins per day.
	- Every minute, 571 new websites are launched.
Google+	- 1 billion accounts have been created.
Google	- The site had over 5.2 billion daily searches in 2017.
	- Every day, 25 petabytes are processed.
Apple	- Approximately 47,000 applications are downloaded per minute.
Amazon	- USD373 million in sales every day in 2017.
Linkedln	- 2.1 million groups have been created.
Email	- Every day in 2017, 269 billion emails have been sent.
Mobile Device Data	- In 2017, 8 exabytes of data have been created on mobile devices.
	- In 2017, there were 3.394 billion mobile Internet users.
Data created by the Internet of Things (IoT)	- 2.5 quintillion bytes of data are created every day by mobile devices, WSNs, Smart TVs, cars, etc.

**Table 2:** Comparison of different data-collection architectures.

Architecture	Reliability	Number of Sinks	Energy Consumption	Latency	Lifespan	Scalability
*Static sensors and sinks*						
Li et al. (2018) [85]	high	many	low	medium	low	yes
Ari et al. (2018) [100]	high	1	low	medium	medium	yes
Wang et al. (2018) [82]	medium	1	medium	high	medium	yes
Ari et al. (2016) [36]	high	many	low	medium	low	yes
Zhang et al. (2012) [101]	low	1	strong	high	low	difficult
Di Francesco et al. (2011) [25]	medium	many	medium	low	medium	yes
Chen et al. (2009) [81]	high	many	medium	low	high	yes
Werner et al. (2006) [47]	low	1	strong	high	low	difficult
*Mobile sensors and static sink*					
Irish et al. (2019) [97]	medium	many	low	medium		no
Handcock et al. (2009) [95]	medium	many	low	medium		no
Huang et al. (2005) [96]	medium	1	medium	high	medium	no
*Mobile sink and static sensor nodes*					
Sabor et al. (2018) [91]	medium	many	medium	medium	high	yes
Kumar et al. (2018) [99]	medium	1	medium	high	medium	yes
Zhong and Ruan (2018) [98]	low	many	medium	medium	medium	no
Ari et al. (2017) [26]	high	1	medium	medium	high	yes
Khan et al. (2013) [102]	high	1	medium	medium	medium	yes
Zungeru et al. (2012) [103]	high	many	medium	low	medium	yes
Jea et al. (2005) [104]	low	1	medium	medium	high	no
Juang et al. (2002) [84]	low	1	medium	low	medium	no
*Hybrid*					
Juang et al. (2002) [84]		depends	weak			no
